# The Needs and Barriers of Medication-Taking Self-Efficacy Among Poststroke Patients: Qualitative Study

**DOI:** 10.2196/14399

**Published:** 2019-07-22

**Authors:** Jamuna Rani Appalasamy, Pathmavathi Subramanian, Kit Mun Tan, Siva Seeta Ramaiah, Joyce Pauline Joseph, Siew Siang Chua

**Affiliations:** 1 Jeffrey Cheah School of Medicine and Health Sciences Monash University Bandar Sunway Malaysia; 2 Faculty of Medicine University of Malaya Kuala Lumpur Malaysia; 3 Department of Medicine Faculty of Medicine University of Malaya Kuala Lumpur Malaysia; 4 Medical Department Subang Jaya Medical Centre Subang Jaya Malaysia; 5 Neurology Department Hospital Kuala Lumpur Kuala Lumpur Malaysia; 6 Faculty of Health & Medical Sciences Taylor's University Subang Jaya Malaysia

**Keywords:** poststroke, medication taking self-efficacy, medication adherence

## Abstract

**Background:**

Stroke is one of the top 10 leading diseases worldwide, with high mortality and morbidity rates. There is an incomplete understanding of the various types of self-efficacy involved in the prevention of recurrent stroke, and one of them is medication-taking self-efficacy.

**Objective:**

This study aimed to explore the fundamental needs and barriers of medication-taking self-efficacy in poststroke patients in Malaysia.

**Methods:**

We performed in-depth individual interviews with poststroke patients (N=10) from the Outpatient Neurology Clinic, Hospital Kuala Lumpur. All interviews were transcribed verbatim, and an inductive thematic analysis was performed on the data collected from the interviews.

**Results:**

Two key themes were identified: (1) self-efficacy in taking the effort to understand stroke and its preventative treatment for recurrent stroke and (2) self-efficacy in taking prescribed medication to prevent stroke. Patients needed to be proactive in seeking reliable information about stroke and the perceived benefits of preventative treatment for stroke. The discussion was focused on eliciting the needs and barriers related to medication-taking self-efficacy. Patients needed to develop independence and self-reliance to overcome barriers such as dependency and low motivation. External factors such as limited information resources, low perceived severity, poor social environment, and poor communication add to the challenges of poststroke patients to improve their self-efficacy of managing their medications.

**Conclusions:**

The study identified potential key findings related to the needs of patients in a localized setting, which are also related to several health behavioral concepts and constructs, indicating the importance of overcoming barriers to improve the quality of life in poststroke patients. We anticipate that the results will be taken into consideration for future personalized patient education interventions.

## Introduction

Surviving a stroke can be an enduring challenge that affects many facets of a person’s life. Stroke was the second leading cause of noncommunicable disease death globally in 2016 [1]. In fact, stroke has been known to be a cause of substantial disability and debility, and its prevalence is estimated to double by 2035 [1,2]. Research on stroke preventative medication such as antiplatelets and anticoagulants by pharmaceutical industries and treatment by health care providers has resulted in outstanding improvements in the morbidity and mortality of poststroke patients [3-5]. Hence, adherence to lifesaving therapies needs to be sustained in order to achieve optimal treatment outcomes. Otherwise, medication nonadherence would result in deficiency of treatment optimization, increasing the risk of stroke and leading to a possibility of stroke recurrence [6-8]. A review paper suggested that poor medication adherence is associated with modifiable patient factors that are related to a lack of understanding caused by low health literacy. The paper also highlighted variances in belief, attitude, and motivation caused by behavioral factors. Poor adherence is also associated with an increase in medication-related problems [6,9]. In other studies, poor adherence of stroke preventative medications among poststroke patients was also associated with a higher prevalence of cognitive disability, depression, low motivation, less social support, and low self-efficacy [10,11]. Interestingly, similar studies have shown a lack of self-efficacy among stroke survivors despite patient education and counseling effort, regardless of various health care settings [12,13]. Patient empowerment was suggested to be a significant facilitator of enhanced medication adherence [6]. Therefore, exploring the effect of self-efficacy on medication-taking behavior was considered to be important.

Self-efficacy is defined as the belief in one’s ability to execute a specific task or actions in order to achieve a goal [14]. This type of faith in oneself leads to high confidence and better control, which translates the intention to perform into carrying out the planned action. Self-efficacy is an important component in various behavioral models such as self-efficacy theory, social cognitive theory, and the health belief model (HBM) and is associated with better medication adherence among poststroke patients [15-17]. However, exploratory research examining the types of self-efficacy underpinning medication-taking behavior among poststroke patients is limited. Identification of the needs and barriers of specific medication-taking self-efficacy is of utmost importance for the development of potential interventions to improve medication adherence. Hence, it is crucial to understand the view and impact of those needs and barriers on poststroke patients. Therefore, the research question of this study is as follows: What are poststroke patients’ needs and barriers to sustain medication-taking self-efficacy? The current qualitative study described findings from individual in-depth interviews of poststroke patients.

## Methods

### Ethics Approval

The design and conduct of the study were approved by the Malaysian Medical Research and Ethics Committee (NMRR ID-15-851-24737) in July 2015.

### Study Setting

The study recruited patients followed up at the Outpatient Neurology facility at Hospital Kuala Lumpur (HKL), who were informed about the study and provided consent. HKL is the principle tertiary facility in Malaysia and receives a high number of patients with stroke from different territories in the Klang Valley and throughout Malaysia, with approximately 1000-1200 acute and recurrent stroke cases every year. The Neurology Department is a pioneer in setting up an acute stroke center in Malaysia, which is overseen by a group of neurologists, doctors, and other health services supporting staff.

### Recruiting Participants

For the interview, patients were randomly selected via a simple random sampling method, from a list of 89 patients with potential drug-related problems (DRP) [18], who were identified by a clinical pharmacist. DRP is a set of categories of medication issues used by medical personnel to conduct a strategic medication review in order to ensure optimization of the prescribed medication. The issues could be related to medication interactions, dosage appropriateness, adverse events, or adherence. Thus, a patient with DRP would be the best candidate to analyze medication-taking behavior [9]. The inclusion criteria were diagnosis with first stroke in the last 6 months from the initial date of screening and interview (January 1, 2016, until March 30, 2016); taking stroke-prevention medications such as statins, antiplatelets, or anticoagulants; and no memory problems and ability to converse, read, and write in Malay or English. We selected patients who had potential treatment issues such as a subtherapeutic effect with causes related to medication use process (eg, medication not taken). In-depth interviews were deemed most appropriate to build a trustful rapport between the researcher and patients and obtain more comprehensive views. This method also ensures that patients are comfortable and that their thoughts are not suppressed due to the presence of other patients. It was not possible to conduct a focus group discussion due to schedule and venue problems, as these patients had physical immobility or transportation issues. One researcher approached all patients and explained the purpose of the interview and study. The research team agreed to recruit more patients until data saturation [19]. However, of the 32 patients who showed interest, only 10 patients (31.2%) agreed and signed the consent form to volunteer for a personal interview for a maximum of 30 minutes. We completed full semistructured interviews with a total of 10 patients (5 women, 5 men; age range: 44-78 years; Table 1).

**Table 1:** Characteristics of the poststroke participants (N=10).

Characteristics		Value
Age (years), mean (SD)		57 (10.01)
**Sex, n (%)**		
	Male	5 (50)
	Female	5 (50)
**Ethnicity, n (%)**		
	Malay	7 (70)
	Indian	2 (20)
	Chinese	1 (10)
**Type of stroke, n (%)**		
	Ischemic	8 (80)
	Hemorrhagic	2 (20)
Number of prescribed medications, mean (SD)		3.7 (0.94)
Medication nonadherence, mean (SD)		2.8 (0.63)
**Employment status, n (%)**		
	Employed	6 (60)
	Unemployed	4 (40)
**Education level, n (%)**		
	Primary	2 (20)
	Secondary	6 (60)
	Tertiary	2 (20)

### Data Collection

In-depth interviews were conducted on the basis of a qualitative open-ended interview guide developed by a nurse educationist, two pharmacist educationists, and a neurologist. This group represented views from different health care professionals involved in stroke treatment and care. The interview guide was pretested on nine poststroke patients for their relevance and suitability in the Outpatient Neurology clinic setting. Although short, the guide was precise in order to prevent burdening patients with many questions while giving them enough time to recall meaningful events (Multimedia Appendix 1). Each patient was given an appointment for the interview. One researcher (JA) led each scheduled participant to a quiet room at the Outpatient Neurology clinic, started the interview after a friendly chat, and continued to brief them about the study. The interviews focused on patient’s experiences of stroke and medication management. The interview was conducted at a relaxed pace, whereby the patient was allowed to pause or have a short break. The researcher (JA) posed prompts whenever necessary or when the conversation was mixed. After 15 minutes of interview, two online video vignettes were shown to the patients. The video vignettes of 2 minutes each were in an animated form, conveying messages on the importance of understanding the disease and its preventative medication and how people perceived their medications (Multimedia Appendix 2). The researchers (JA and SR) developed the video vignettes in English and Malay language to prompt patients to elicit a deeper thought of self-reflection, and in doing so, the video allowed the researchers to obtain a better understanding of the patients’ needs or barriers to improve their health conditions. Vignettes were suitable, as they empowered accumulation of delicate subjective information and are a successful device for inspiring judgments and discernments [20]. The videos that lasted less than 2 minutes had satisfactory face validity and content validity, as confirmed by three poststroke patients, a clinical nurse, two neurologists, and two pharmacists. Patients were asked to describe their experiences of adhering to prescribed medications after viewing the video. All responses were audio recorded, and the researchers ensured the confidentiality of the recordings.

### Data Analysis

All interviews were transcribed verbatim, checked by another researcher, and then documented for data analysis. All transcripts were manually coded and classified using the inductive thematic analysis methodology [21]. The methodology were as follows: (1) understanding the transcripts, (2) diagramming key points into codes, and (3) summarizing the mapped codes into subthemes and emergence of major themes. Six scripts in the Malay language were translated by an independent translator. Two researchers (JA and SR) reviewed the transcripts and met intermittently to discuss the themes, outlines, and issues established in the data. Once the themes were generalized, they were verified by two other reviewers (PW and CS) to ensure uniformity, precision, and quality [22].

## Results

### Study Themes

Two major themes related to medication-taking self-efficacy were identified: self-efficacy in taking the effort to understand stroke and its preventative treatment for recurrent stroke and self-efficacy in taking prescribed medications to prevent stroke. A majority of the subthemes discussed by the patients referred to the individuals’ necessity, needs to attain the specific self-efficacy, and the challenges considered to be barriers to attaining those needs. Thus, the results section is organized into two sections that discuss the needs and barriers of both main themes. The first portion of each section discusses the needs and the second portion discusses the barriers. The themes emphasized on individual well-being, communication, and independence, which were elicited in response to specific probing of the elements related to medication-taking self-efficacy.

### Self-Efficacy in Taking the Effort to Understand Stroke and its Preventative Treatment for Recurrent Stroke

#### The Needs: Proactive in Acquiring Information

Self-efficacy is a necessity that determines how one approaches challenges and accomplishes tasks. It is a belief in oneself to be able to achieve planned goals. The needs of poststroke patients in this study related to medication-taking self-efficacy were concluded based on deduction from specific cues, quotes, and observed expressions. The poststroke patients in this study recognized that they required the confidence to be “independent and active learners” in order to gain knowledge about stroke recurrence and rationalize how the stroke occurred and why they must adhere to preventative medications. In other words, patients would need to be responsible for taking their own decisions and their own efforts to seek more information about their illness and treatment rather than accepting any passive information. One patient stated:

Once you have a stroke, you need to read a lot to know more about it…we don’t know when we can get it again.

ID3

By doing so, the patients were able to justify the importance of taking prescribed medications and improving their adherence to treatment:

We need to take the medicine, if not it can worsen our condition; that’s what I’ve learnt from the internet.

ID8

#### The Needs: Perceived Benefit of Stroke and its Prevention Therapy

We assumed that a minority of patients lacked the trust and belief in information, which was crucial to guide their actions toward understanding the purpose of adhering to prescribed medications, as exemplified by the following statement:

I don’t know why the doctor gave me so much medicine...I take the doctor’s medicine alternately because I can control my blood pressure with my own herb mixture.

ID2

This statement reflects a current situation wherein patients had the tendency of negative belief, which gave them an impression that their medication-taking actions were more beneficial than the advised information. Hence, there is a need to increase patients’ awareness to appraise any information about their illness and its treatment regardless of the source of information or belief.

#### The Barriers: Limitation on Reliable Information Resources

The patients also expressed some disappointment in not receiving vital information about stroke on time before the actual event occurred.

I only learned more about stroke when I got one.

ID7

The information about stroke and its prevention could have been delivered in various media formats or oral communication. One patient’s statement strongly indicated the existence of the barriers:

I didn’t know I was having stroke, until my daughter explained to me.

ID3

Participants acknowledged that health care clinics’ efforts to educate patients are important, but they were also concerned about the limited resources or health care facilities for obtaining information, particularly on optimizing treatment for personal benefit.

I know they (the doctors) are very busy. So they don’t have time to explain.

ID9

I can’t remember everything…I think they should give us free medicine box.

ID10

#### The Barriers: Poor Communication

There were possibilities of poor communication or language barriers between the prescriber and patient. This issue was potentially related to the lack of self-efficacy in taking the effort to understand the purpose of stroke preventative medication:

Sometimes, I don’t understand what the doctor or pharmacist told me.

ID5

#### The Barriers: Lack of Perceived Severity

A diversity of perception was identified from spontaneous remarks by the patients. Low perceived severity occurs when there is a low inclination toward acceptance of illness, that is, belief that a stroke is not a serious disease. One patient lamented:

I know I was having some symptoms…but I felt it was ok so I kept on driving because it went away after a while.

ID2

Poststroke patients showed negative perception of the value of stroke-prevention medication, particularly antiplatelets, anticoagulants, and antihypertensive agents. Patients perceived a lack of benefit of these prescribed medications and misconceptions of their mechanism of action:

The medicine will definitely cause more side effect…it is toxic especially to your kidneys...you just need to relax to bring down the blood pressure, sometimes I control it myself.

ID4

#### The Barriers: Environmental Influence on Medication-Taking Behavior

Patients’ environment and experiences acted as barricades (excellent influence) for perceived illness and medication-taking self-efficacy of prescribed medications. Family, friends, and common health practice in a community influenced these patients’ attitude and action of responsibility toward their illness, which affected their medication-taking behavior:

My friend bought me this tea, it thins your blood…if I tell my doctor, he will definitely disagree...but I know it works.

ID1

My children and neighbor asked me to try some herbs. You know that expensive one for blood circulation...but I don’t want to...

ID7

There was also influence from electronic media, which acted as a stimulus of behavior changes and action:

I learn a lot using the internet, this person advises you can take certain herb, so I tried it out.

ID8

These phenomena challenge the patient education efforts made by public agencies and hinder their efforts for instilling positive medication-taking behavior among poststroke patients.

### Self-Efficacy in Taking Prescribed Medication to Prevent Stroke

#### The Needs: Independence and Self-Reliance

One of the underlying reasons for not being adherent is the lack of independence in medication taking and self-management. Poststroke patients realized that independence and self-reliance have a positive effect on managing prescribed medications, and this is one-step toward success in improving their stroke conditions.

I googled more...you need to know what and how you take your medicine. I asked the doctor about my medicine if I don’t understand.

ID3

Nevertheless, poststroke patients need to have confidence and trust that their prescribed medications will benefit them, albeit the acceptable risks:

We should not be afraid of side effect, you have no choice but to take it...because the medicine benefits you.

ID8

#### The Barriers: Dependency in Medication Management

Success in managing one’s own medication requires ample skills and perseverance to overcome obstacles. However, this “mastery experience” could also undermine self-efficacy belief if failures were not overcome, which in turn become a norm and increased dependency. A few patients provided testimonials on reflecting on a potential failed experience and increasing dependency on managing medications:

I have limited moving ability to manage my medicine, so, my wife takes care of them.

ID6

I don’t know much about the medicine...you have to ask my daughter.

ID5

I was not informed how to store my medicine...they didn’t teach me, but just briefly told me at the counter.

ID2

#### The Barriers: Low Motivation

Despite the need for self-efficacy for taking medications, a lack of motivation has been a challenge for those who wanted to change; hence, this factor is the foremost barrier against self-efficacy toward medication taking and management. Patients expressed feelings of not being understood and suffering alone, and there were high chances that they were getting frustrated and depressed.

Those who didn’t experience stroke, don’t understand how I feel.

ID1

Physical disability is also a huge barrier that complements low motivation.

I tried going for rehab for six months…no improvement, everyone kept advising me the same thing, what is the use?

ID4

Apart from low motivation affecting self-efficacy toward medication adherence, it was evident that there was a transit effect on the patients’ quality of life.

## Discussion

### Principal Findings

Normally, confidence is thought to be adequate to carry out a task, and it is simple to adhere to medications. Confidence has a positive effect on self-efficacy; however, this may not be the case vice-versa. The ability to understand, think, plan, and use prescribed medications to sustain medication adherence and ensure treatment effectiveness depends on the individual’s self-efficacy levels [15,16,23]. Hence, self-efficacy in understanding and taking medication appropriately found its specific niche in nonadherence and has been studied for more than a decade [24]. This qualitative study managed to obtain an overview of poststroke patients’ needs and barriers toward sustainable medication-taking self-efficacy.

Patients expressed a lack of the understanding that every stroke event portrays different symptoms, and inability to control stroke risk factors increases the risk of a recurring stroke event. This finding was consistent with a previous qualitative study of stroke patients’ perception, which highlighted the difficulty of identifying various atypical stroke symptoms [25]. It was clear that the patients were not proactive. About 5 of the 10 patients (50%) knew that relevant knowledge was important, but they were laid-back even though they were experiencing a stroke. Moreover, the lack of knowledge and awareness was limited to not only illness but also its preventative treatment. A trend of negative responses from those with a lower health literacy level was observed: 2 of the 10 patients (20%) had poor literacy levels. To achieve self-efficacy in order to gain an understanding of stroke and its preventative medication purpose, patients were faced with barriers such as inadequate or unreliable sources of information and poor communication with prescribers, which dampen their knowledge search. Importantly, unassessed low health literacy complements the knowledge attainment barriers [25].

Other than being proactive, perception and belief are essential in the patients’ decision making process in medication-taking behavior. Perception is subjective of what an individual thinks about an issue and how one is influenced by one’s beliefs. Thus, the intensity of medication-taking self-efficacy depends on the level of patients’ perception of their disease and its treatment [17,26]. Therefore, we could summarize that patients with similar underlying stroke risk factors or severity may not have the same perception of illness and belief about their medications even if they were assumed to have the same knowledge about stroke and its treatment. It is possible that varying physical and emotional experiences with stroke cause differences in perception and beliefs. Therefore, actions of poststroke patients’ toward adherence are steered by their perceived susceptibility and severity of stroke and led by their perceived benefits or barriers of medication taking, which are influenced by their beliefs [27,28]. Nevertheless, diverse populations and cultures have a strong influence on these beliefs too [29,30]. Hence, the success of a behavioral intervention depends on these factors as well.

Self-efficacious patients tend to develop or learn skills on their own in an effort to overcome worsening of the disease condition. They choose self-empowerment and a high responsibility of medication management to ensure optimal therapy effect. Some self-care examples are monitoring blood parameter, scheduling medication intake using the pill reminder, preparing appropriate medicine storage, and being aware of any allergy symptoms [31-33]. In the same way, self-efficacy is highly influenced by parallel changes in self-motivation and quality of life, and these changes vary between individuals according to the effect of personal experiences (mastery experience) and the perspectives of stroke and belief in the preventive treatment [12,34,35]. In other words, self-efficacious patients living with long-term illnesses tend to put effort into attaining an in-depth knowledge about their disease and treatment, creatively solving problems, and increasing their expertise of improving their own health [36]. Thus, in order to sustain medication-taking self-efficacy for optimizing treatment effectiveness, health professionals should ensure that their intervention fulfils patients’ needs of self-efficacy to understand and use medication appropriately and that it addresses their barriers toward the intended self-efficacy.

### Study Limitation and Strength

This study was based on the viewpoints of 10 poststroke patients. We experienced a high drop-out rate, as we were unable to secure appropriate funding when patients requested for their financial reimbursement. Therefore, generalizability of the study findings was limited by the small sample size. There were also challenges in distinguishing the needs and barriers of language or health literacy level. Hence, more in-depth interviews with validated tools are required to categorize varying health literacy levels among poststroke patients. This sample may not have captured the full range of the needs and barriers of poststroke patients. However, this did not detract from the clear importance of the two main needs of medication-taking self-efficacy: self-efficacy in gaining an understanding of stroke and managing the intake of preventative medications for stroke.

The strength of this study is that it revealed emotional experiences of poststroke patients under an unconstrained technique with the help of focused prompts and video vignettes to elicit deeper thoughts compared to close-ended questionnaires. The themes were discussed in specific probes of self-efficacy related to medication adherence dimensions. Hence, this study indirectly proposed the usefulness of video in modifying focused or planned behavior [37,38].

### Conclusions

Our findings were exploratory, and hence, the outcomes on verifying the association between the needs and barriers and medication-taking self-efficacy should be confirmed using quantitative measures with adequate sample size. Nevertheless, the results provided a subjective perspective of poststroke patients based on their experiences, and thus, it is crucial to consider these viewpoints as a groundwork for future interventions related to understanding medication taking and its self-efficacy.

## Appendix

Multimedia Appendix 1 Patient interview guide.

Multimedia Appendix 2 Video 1.

Multimedia Appendix 3 Video 2.

## Abbreviations

DRP: drug-related problems
HBM: Health Belief Model
HKL: Hospital Kuala Lumpur
