# Supplementary material for: The Needs and Barriers of Medication-Taking Self-Efficacy Among Poststroke Patients: Qualitative Study
Source: JMIR Nurs. 2019 Jul 22;2(1):e14399. doi: 10.2196/14399 (PMC8279437; doi:10.2196/14399)
Supplement: Multimedia Appendix 1 [file nursing_v2i1e14399_app1.docx]

Semi-structured interview guide.

**the word ‘medicine’ could be replaced with the names of medicine that the patients are taking currently e.g. Aspirin, Plavix…(information obtained from their medical records prior the interview)

Good morning/ afternoon.

Thank you for taking the time to meet with me and for the audio recording consent. I would like to ask you several questions about your medicines.

I will ensure that all the information we collect are to be kept confidential. Do you have any questions before we begin? There is no obligations to answer all questions. You may stop or leave the interview at any time.

Please feel free to ask questions at any time during our conversation. Let’s get started;

1. Please describe about your stroke experience.

Prompts: How do you feel?

2. What do you think about your prescribed medicine?

Prompts: Tell me your belief about its medicinal value?

3. How do you follow a schedule/ take your medicine?

Prompts: Can you show/ demonstrate/describe

4. (After watching the video) In your opinion, what would you think about your medication adherence?

Prompts: Briefly describe you experiences

Thank you for your insightful responses to my questions.
